# Locomotion and eating behavior changes in Yucatan minipigs after unilateral radio-induced ablation of the caudate nucleus

**DOI:** 10.1038/s41598-019-53518-2

**Published:** 2019-11-19

**Authors:** Nicolas Coquery, Jean-François Adam, Christian Nemoz, Régis Janvier, Jayde Livingstone, Alain Chauvin, Samy Kefs, Cécile Guerineau, Loic De Saint Jean, Alexandre Ocadiz, Audrey Bouchet, Stefan Bartzsch, Elisabeth Schültke, Albert Siegbahn, Elke Bräuer-Krisch, Benjamin Lemasson, Emmanuel Luc Barbier, Jean Laissue, Jacques Balosso, David Val-Laillet, Raphael Serduc

**Affiliations:** 1 INRA, INSERM, Univ Rennes, Nutrition Metabolisms and Cancer, NuMeCan, Rennes, St Gilles France; 2 Inserm UA7, Rayonnement Synchrotron pour la Recherche Biomédicale (STROBE) Université Grenoble Alpes – ID17, Installation Européenne du Rayonnement Synchrotron (ESRF) CS, 40220 Grenoble, France; 3 Centre Hospitalier Universitaire Grenoble-Alpes, La Tronche, France; 4 European Synchrotron Radiation Facility (ESRF), Grenoble, France; 5 Australian Synchrotron, Clayton, Melbourne Australia; 6 INRA, UEPR, Saint-Gilles, France; 7 Technical University of Munich, School of Medicine, Klinikum rechts der Isar, Department for Radiation Oncology, Munich, Germany; 8 Helmholtz Zentrum München, Institute of Radiation Medicine (IRM), München, Germany; 9 Department of Radiooncology, Rostock University Medical Center, Rostock, Germany; 10 Department of Clinical Science and Education, Karolinska Institute, Stockholm, Sweden; 11 Grenoble Institut des Neurosciences, InsermU1216, University Grenoble Alpes, Grenoble, France; 12 University of Bern, Bern, Switzerland

**Keywords:** Neuroscience, Brain injuries

## Abstract

The functional roles of the Caudate nucleus (Cd) are well known. Selective Cd lesions can be found in neurological disorders. However, little is known about the dynamics of the behavioral changes during progressive Cd ablation. Current stereotactic radiosurgery technologies allow the progressive ablation of a brain region with limited adverse effects in surrounding normal tissues. This could be of high interest for the study of the modified behavioral functions in relation with the degree of impairment of the brain structures. Using hypofractionated stereotactic radiotherapy combined with synchrotron microbeam radiation, we investigated, during one year after irradiation, the effects of unilateral radio-ablation of the right Cd on the behavior of Yucatan minipigs. The right Cd was irradiated to a minimal dose of 35.5 Gy delivered in three fractions. MRI-based morphological brain integrity and behavioral functions, *i.e*. locomotion, motivation/hedonism were assessed. We detected a progressive radio-necrosis leading to a quasi-total ablation one year after irradiation, with an additional alteration of surrounding areas. Transitory changes in the motivation/hedonism were firstly detected, then on locomotion, suggesting the influence of different compensatory mechanisms depending on the functions related to Cd and possibly some surrounding areas. We concluded that early behavioral changes related to eating functions are relevant markers for the early detection of ongoing lesions occurring in Cd-related neurological disorders.

## Introduction

The function of the Caudate nucleus (Cd) has been extensively studied. This yielded a good knowledge of its involvement in several brain functions^1^, such as in motor function, memory^2^, motivation, hedonism, and emotion^3^. Intentionally disturbed Cd functions have been investigated for basic research purposes. Selective Cd lesion can also occur in brain pathologies and cause different degrees of invalidation, from partial dysfunction in Huntington disease^4^ to total invalidation such as in stroke^5^. Ablation of the Cd has also been investigated as a therapy for brain pathologies such as Parkinson’s disease^6^ and movement disorders^7^. However, little is known about the behavioral changes impacting Cd-related brain functions, which can be induced along with a progressive ablation of the Cd.

Compared to traditional neurosurgery, technologies such as stereotactic radiosurgery (SRS) made possible minimally invasive procedures aiming at the limitation of tissue destruction to the region of interest (ROI), *i.e*. the targeted brain region. SRS is indeed well suited for targeted cerebral destruction^8^ while sparing non-targeted brain parenchyma^9^. Among the SRS approaches, the Gamma knife has been extensively used for treating neurological diseases that interfere with function, and the indications include movement disorders (tremor), brain tumors such as metastatic melanoma, arteriovenous malformations, and pharmacoresistant epilepsy^10^. Alternative approaches are also under development, such as focused ultrasound, which has shown favorable results in pigs^11^. SRS is also currently proposed as a promising approach for neuromodulation^12^, which could ultimately be used in the therapy for patients with psychiatric disorders^13^ as a component of the growing concept of “surgery of the mind”^14^. SRS does not promote an instantaneous ablation of the targeted brain structures, as compared with focused ultrasound- and radiofrequency-based ablation^11^. Thus, SRS can be a useful tool to study brain structures with different levels of invalidation, and ultimately, to investigate the compensatory processes involved in the preservation of the impacted functions.

Synchrotron radiation exhibits interesting features for SRS, such as high-flux quasi parallel x-rays^15^. These unique properties combined with spatial fractionation (arrays of microbeams) offer dose gradients about 200 times steeper than that of conventional radiotherapy and might allow extremely precise delivery of high ablative doses of radiation deep in the brain, within short exposure times, and without affecting adjacent normal tissues^16^. The use of synchrotron radiation based SRS in medium-size animals is however in his premises^17^, even if large data is available on rodent models^18^. In this study, we combined synchrotron-generated X-ray microbeams radiosurgery, *i.e*. microbeams radiotherapy (MRT), with “conventional” hypo fractionated radiotherapy, *i.e*. stereotactic radiotherapy (SRT) delivered by a 6MV Linac. The choice of delivering the dose in three fractions, compared to one in SRS, was made to stay on the safe side in terms of healthy tissue complications for this first trial. Moreover, as it was planed to use synchrotron as a radiation boost in conventional hypofractionated radiotherapy in future oncology trials, the data acquired during this experiment are of particular relevance for planning future clinical trials^19,20^.

Medium-size animals, such as (mini)pigs, are currently gaining interest as preclinical models in translational research with the aim to perform further human clinical trials. From an animal ethics perspective, (mini)pigs can be considered as an alternative to primate models. Many mental and behavioral disorders can be modeled in such larger animals^21^. The minipig model is increasingly used as a preclinical model of neurological disorders, such as for Parkinson’s disease^22^ and glioblastoma^23^. Their brain anatomy and size are also well suited for the use of brain imaging and the development of therapies to be tested in human clinical trials^24^. For instance, targeted ablation of a specific brain region has been efficiently implemented in pigs and minipigs^11,25^.

Here, we aimed at providing a detailed behavioral description of a progressive radio-ablation of the Cd. For this purpose, we performed a stereotactic ablation of the right Cd in Yucatan minipigs with a combination of hypofractionated SRT and MRT^26^. Over a period of twelve months, we followed the kinetics of (i) development of the radiation-induced lesion-ablation using anatomical MRI images and, (ii) the Cd-related brain function modifications observed as motor functions changes scored in the openfield (OF) test, and as motivation/hedonism determined in eating behavior tests. We assumed that the Cd-related functions would be modified with different kinetics during the ongoing ablation of the Cd, due to different function-dependent compensatory processes. The results of our study suggest that the first detected behavioral change should be of high interest as a sentinel signal, a diagnostic marker for the early detection of neurological disorders involving the Cd.

## Results

### Body weight evolution

**Figure 1:**
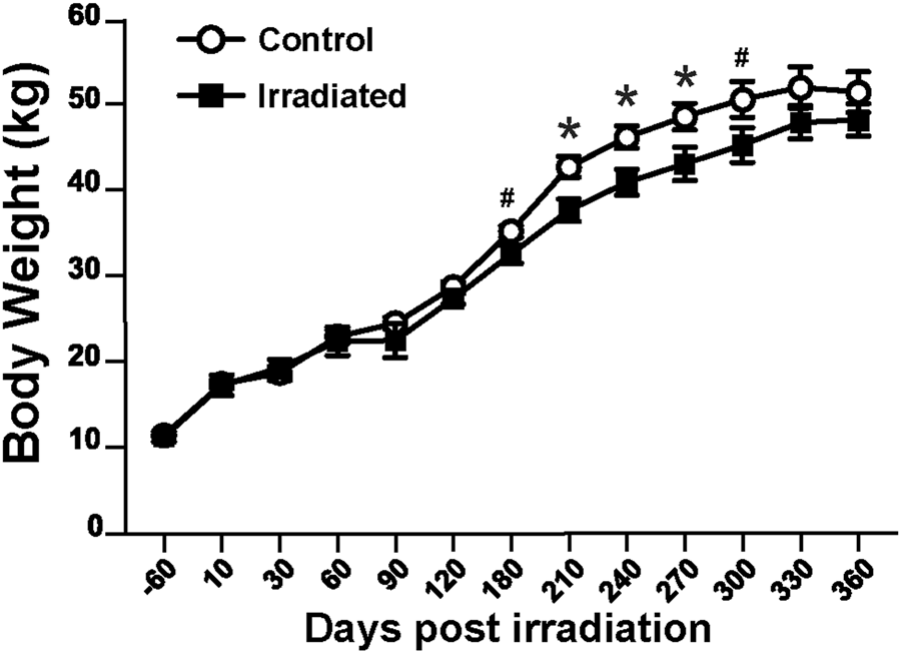
Animals’ body weight: days before (−60) and after irradiation on Day 0. Mean ± SEM. ANOVA, *p < 0.05; # 0.05 < p < 0.10.

Control animals showed an increase in body weight along the entire study period, with a growth curve typical of Yucatan minipigs of this age (between 6-month and 1.5-year-old) (Fig. 1). The body weight increase of irradiated animals slowed down between Day 60 and 90 after irradiation even though the change was not statistically significant compared with control animals. Between 90 and 210 days after irradiation, the body weight of the irradiated animals increased less than the weight of control animals, leading to a statistically significant difference 210 days after irradiation (p = 0.009) that persisted until Day 270 after irradiation. The difference was then reduced until both groups had similar weights 330 days after irradiation (p > 0.1).

### Caudate (Cd) lesion establishment after irradiation

**Figure 2:**
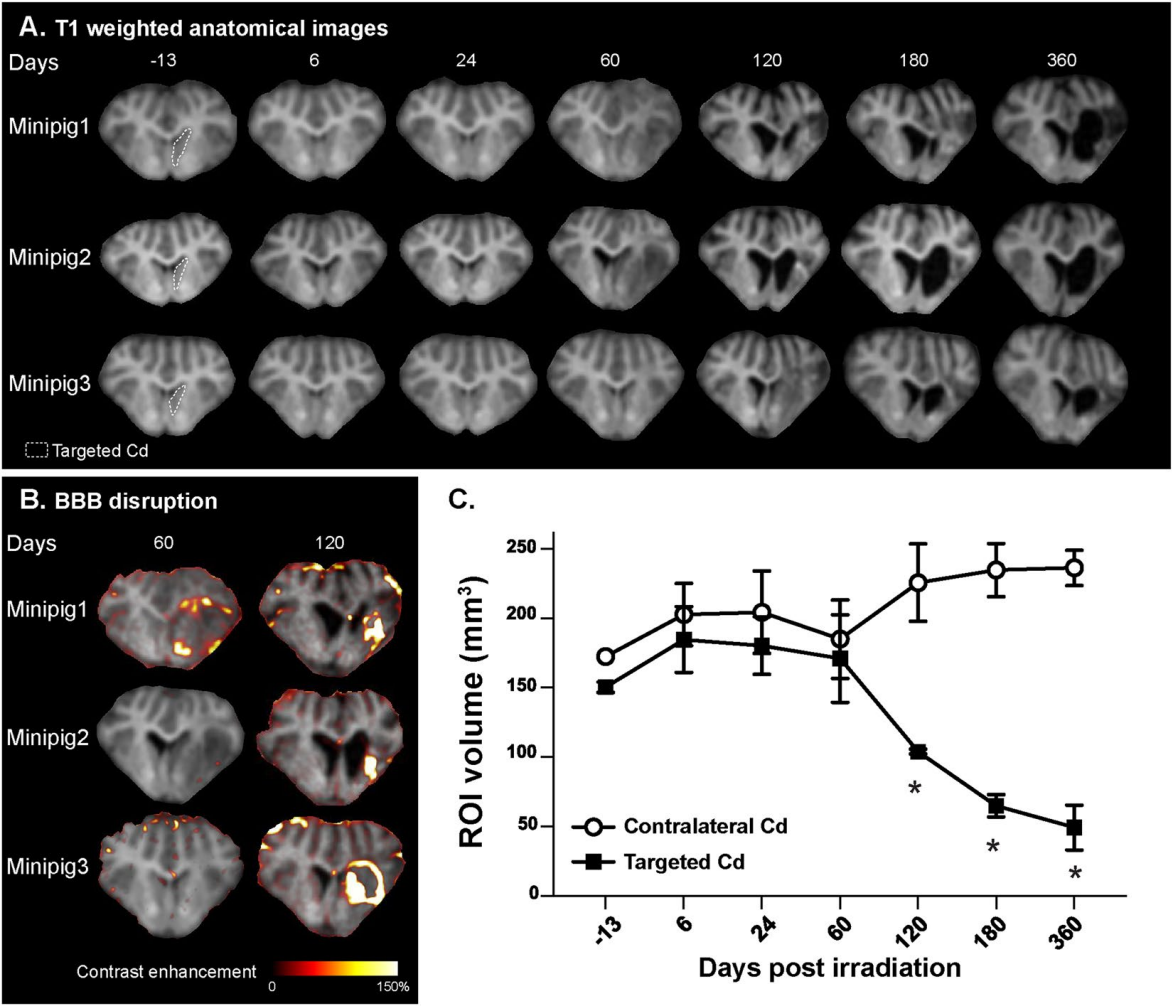
(**A**) Anatomical T1 weighted images for the three irradiated animals before (−13 days) and after irradiation (6, 24, 60, 120, 180 and 360 days). The targeted caudate nucleus (Cd) is shown inside the white dotted line (at −13 days). (**B**) Blood brain barrier (BBB) disruptions for the three irradiated animals were determined 60 and 120 days after irradiation by T1 weighted-based contrast enhancement (in color), after Gd-DOTA intravenous injection. Contrast enhancement is superimposed on T1 weighted anatomical images. (**C**) Two measured regions of interest (ROI) volumes (mm^3^): targeted and contralateral Cd, performed on the T1 weighted anatomical images before (−13 days) and after irradiation (9, 24, 60, 120, 180, 360 days). Mean ± SEM. ANOVA, *p < 0.05.

At visual inspection of MR images, the brain parenchyma of the irradiated animals remained unchanged until 24 days after irradiation (Fig. 2A). Sixty days after irradiation, a hyposignal was detected in the ipsilateral hemisphere in two minipigs (minipigs 1 and 2), including the targeted Cd and the insular cortex. High signal originating from white matter tracts was also reduced, such as in the cortex (minipig 1) and in the *capsula interna* (minipigs 1 and 2). From Day 120 to 360 after irradiation, the targeted Cd and the surrounding right hemispheric brain tissues volumes decreased concomitantly while the volume of both lateral ventricles increased (hydrocephalus, much more pronounced on the irradiated side). After Gd-DOTA intravenous injection, we observed a moderate, low and marked contrast enhancement 120 days after irradiation in the targeted Cd of minipigs 1, 2 and 3, respectively (Fig. 2B).

Two regions of interest (ROIs) were drawn to delineate the targeted and contralateral Cd, and the ROIs volumes (mm^3^) were computed (Fig. 2C). Until 60 days after irradiation, the ROI volume of the targeted Cd remained similar to that of the contralateral Cd. Between Day 60 and 120 after irradiation, the ROI volume of the targeted Cd significantly decreased in comparison to the contralateral reference Cd (Day 120: targeted Cd = 104.0 ± 1.7 mm^3^, contralateral Cd = 225.7 ± 27.8 mm^3^, p = 0.012). The targeted Cd volume decreased until Day 360 after irradiation down to a total reduction of nearly 80% compared with the unirradiated contralateral Cd (Day 360: targeted Cd = 49.3 ± 16.2 mm^3^, contralateral Cd = 236.3 ± 12.7 mm^3^, p = 0.001). The contralateral Cd volume remained stable between all observation times.

### Radiation effects on animal locomotion; openfield test

**Figure 3:**
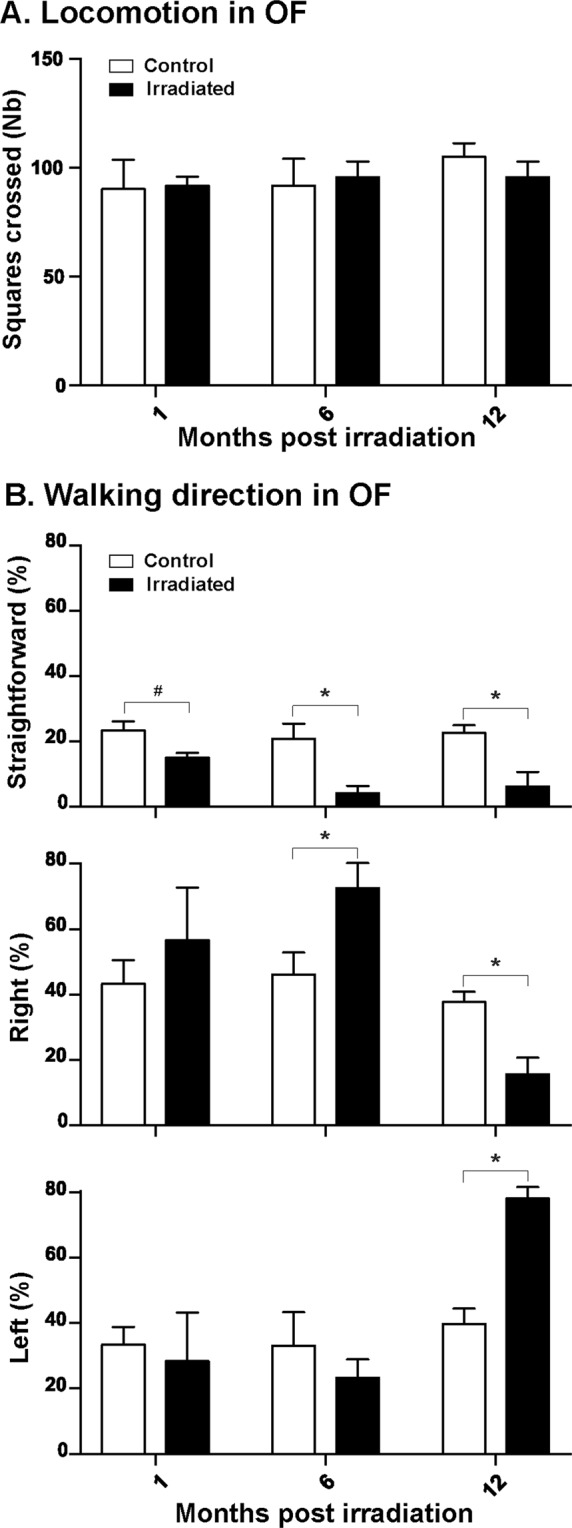
Animal behavior in the openfield after irradiation (1, 6 and 12 months). (**A**) Locomotion determined as the number of virtual squares crossed during the test. (**B**) Walking directions determined as the percentage of walking with continuous sampling of straightforward, right and left walking directions during the test. Mean ± SEM. ANOVA, *p < 0.05; # 0.05 < p < 0.10.

The number of squares crossed in the openfield was similar between irradiated and control animals at 1, 6 and 12 months after irradiation (Fig. 3A). No difference was detected in the time spent immobile.

The percentage of time spent in the center of the openfield was higher for irradiated animals 12 months after irradiation (25.7 ± 2.1%) than in the control group (13.8 ± 2.1%, p = 0.011). The alterations of walking directions in the openfield test are displayed in Fig. 3B. Until one month after exposure, irradiated animals had similar walking characteristics compared to control animals; a tendency toward a decreased straightforward walking was observed in the irradiated group (irradiated group = 15.0 ± 1.4%, control group = 23.4 ± 2.6%, p = 0.062). Six months after irradiation, the irradiated animals showed a significant decrease in straightforward walking compared with control animals (4.1 ± 2.2% *vs*. 20.8 ± 4.6%, respectively; p = 0.034), with increased walking in the right direction (72.6 ± 7.7% *vs*. 46.1 ± 6.7% in the control group, p = 0.049). One year after exposure, walking behavior was modified and irradiated animals showed a reduced straightforward walking (irradiated group = 6.2 ± 4.5%, control group = 22.6 ± 2.3%, p = 0.017) with a decreased walking toward the right direction (irradiated group = 15.6 ± 5.1%, control group = 37.7 ± 3.2%, p = 0.012) and a preferential walking toward the left direction (irradiated group = 78.1 ± 3.5%, control group = 39.8 ± 4.7%, p = 0.002).

### Radiation effects on eating behavior

**Figure 4:**
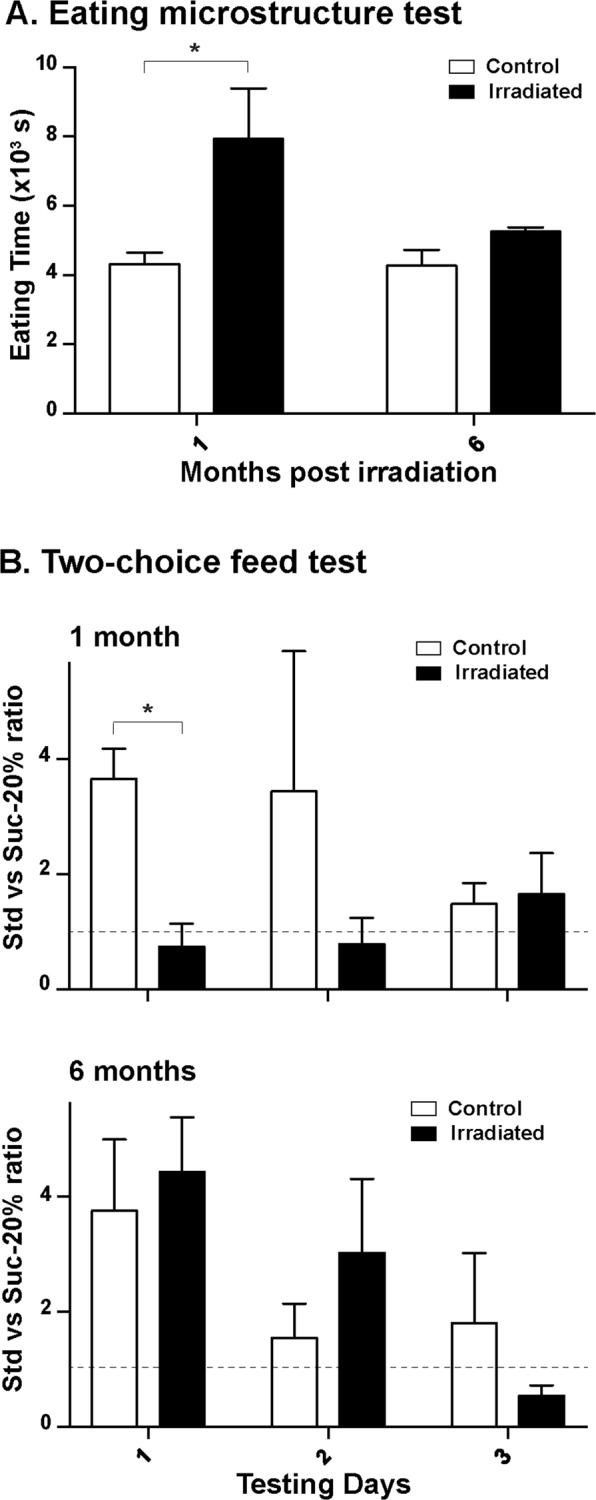
Eating behavior tests after irradiation (1 and 6 months). (**A**) Time to complete a meal of standard feed. (**B**) Feed preference between standard feed (Std) and standard feed with 20%-sucrose (Suc-20%) in the two-choice feed test. Data from three consecutive testing days are presented for each time point after irradiation. Dotted lines refer to a ratio of one, *i.e*. similar consumption of Std and Suc-20% feeds. Mean ± SEM. ANOVA, *p < 0.05.

The eating behavior during a classical meal, *i.e*. one kilogram of standard feed given in one bolus, was modified in irradiated animals from one month after irradiation (Fig. 4A). Irradiated animals took more time to finish their meal in comparison with control animals (irradiated group = 7927 ± 1461 s, control group = 4310 ± 346 s, p = 0.038). The eating time of irradiated animals reverted to the control level six months after irradiation.

A similar effect was observed for the two-choice feed tests: The difference between groups at 1 month was not detectable anymore at 6 months after irradiation (Fig. 4B). At 1 and 6 months after irradiation, control animals tended to prefer the standard feed compared with standard feed + sucrose (20%) during the first testing day. Conversely, one month after irradiation, irradiated animals did not show any neophobia towards the standard feed + sucrose (20%) (first testing day, Std *vs*. Suc-20% ratio: irradiated group = 3.7 ± 0.5, control group = 0.7 ± 0.5, p = 0.012); the ratio of standard feed *vs*. standard feed + sucrose (20%) remained stable and close to the one during the three testing days (Fig. 4B, dotted line. During the three testing days, six months after irradiation, irradiated animals showed similar patterns of preference compared with control animals.

## Discussion

The dynamic effects of a radioablation of the right Cd in the Yucatan minipig, from sub-acute (few weeks) to late phases (1 year)^27^ were characterized in this study, using different endpoints, such as eating behavior, motivation toward food, locomotion and emotional reactivity. Stereotactic ablation of the Cd led to a sub-acute modification of hedonic and/or motivational behavior and motor disturbances, which were detectable before the neurological lesions could be observed on MR images.

The MR characteristics of the lesion were in line with those of a lesion after gamma knife radiosurgery performed in piglets, *i.e*. MR-based lesions were detected as early as 3 months after irradiation^11^. MR images revealed that our irradiation procedures induced neuroradiological changes between Day 60 and 120 after irradiation, when the increase of the body weight of the irradiated animals started to slow down compared with controls. Indeed, between Day 60 and 90, the slope of body-weight curve slowly inflected until the body-weight difference became statistically significant at day 180, *i.e*. six months after exposure. This body weight difference could be the consequence of several early radio-induced alterations: (i) increased energy expenditure through increased physical activity, (ii) increased metabolic expenditure related to inflammation, (iii) reduced food intake due to disturbed eating behavior (the irradiated animals were pooled with control animals in the same cages, which resulted in competition for food access). Because irradiated animals required longer eating times during the microstructure tests, we postulate that the differences in body weight might most likely be explained by differences in eating behavior. At six months after irradiation, the eating behavior of the irradiated animals was similar to that of control minipigs. Afterwards, the slope of body weight curve of the irradiated animals returned to a level comparable to that of the control animals, resulting in similar body weights at the end of the experiment (1 year post-irradiation). The first behavioral radio-induced changes were indeed detected on eating behavior, and especially on both eating microstructure and motivation toward food. Interestingly, both types of behavioral changes were measured one month before observing any morphological changes on anatomical MR images (at 120 days after irradiation). More detailed MRI analyses, such as those used for brain cancer diagnosis, *i.e*. multiparametric microvascular MRI^28^ or magnetic resonance spectroscopy^29^, might yield further insights into ongoing events in this early phase after irradiation.

Only minor effects of radiation exposure on locomotion have been detected one month after irradiation, which corroborates previous studies showing no changes in neurological findings 6 weeks after SRS of the Cd in baboons^30^. Although we could not detect any effects of the irradiation after 6 months on the total distance covered in the openfield, we have measured an increased lateralization toward the right-side direction during walking tests. At this stage, we also observed a stereotypic circling behavior toward the right-side direction in irradiated animals; this is in line with a previous observation performed in a rat model of hemi-parkinsonism^31^. One year after irradiation, the lateralized circling behavior was alleviated but the lateralization was inverted compared with results obtained at 1 and 6 months post irradiation. Indeed, the irradiated animals were walking predominantly towards the left direction, whereas the brain parenchyma and the size of the lesion remained similar to that measured six months after irradiation. We also recorded an increased time spent in the center of the openfield in irradiated animals twelve months after exposure, suggesting a long-term effect on animal emotional reactivity, such as a decreased anxiety-like behavior. Emotive or stressed pigs usually spend more time in the periphery of the openfield arena, which suggests that irradiated animals were less emotive, or had difficulties in interpreting the situation or the environment as distressful. Further investigations using dedicated behavioral tests are needed to investigate the effects of Cd radio-ablation on emotions. Indeed, SRS has been proposed as an innovative strategy against mood-related disorders^14^. The targeted irradiation of the right Cd showed an evolving effect on animal behavior over time, which was not strictly correlated to the presence of lesion in the MRI images. Our results suggest that three mains functions of the Cd are differentially impacted by our combined irradiation protocol: (i) the motor function, assessed during the openfield test, (ii) the hedonism, in relation to the eating microstructure and two-choice feed tests, and (iii) the emotional reactivity in relation to the time spent in the center of the openfield. The onset of symptoms related to these three functions was sequential with (i) a first transitory effect on hedonism one month after irradiation, (ii) a delayed change on motor function six months after irradiation, and (iii) a late effect on motor function and emotion. The differential impact on Cd functions might also be attributed to the individual component of the irradiation, *i.e*. to SRT versus MRT. Additionally, as observed in the MRI images twelve months after irradiation, the lesion was not restricted to the Cd at this time point and was extended to the surrounding tissues, such as the internal capsule. The late effect on motor function might also be attributed to the impairment of this surrounding brain region, and might not be specifically restricted to the Cd invalidation.

The changes in eating behavior one month after irradiation can be attributed to Cd-related hedonic and/or motivational functions because the major homeostatic structure, *i.e*. the hypothalamus, was spared from irradiation. The increased time required for finishing a meal might be either a consequence of decreased motivation or a consequence of motor dysfunction of irradiated animals, for instance mastication. However, as we also found an effect of irradiation in the two-choice feed test, we reckon that the taste perception, which also depends on the insular and gustatory cortices, might have been modified also, in addition to the Cd-related hedonic and/or motivational functions. These effects on eating behavior were alleviated six months after irradiation when the targeted Cd and the ipsilateral insular cortex started to show the first neuroradiological anomalies. This suggests that (i) the regulation of eating brain functions is not specific to the Cd in the right hemisphere and that (ii) a rapid functional compensation from contralateral brain structures can occur to preserve the fundamental functions. The early detection of eating behavior modifications could thus be a useful indicator for diagnosing ongoing neurological lesions that cannot be documented by usual clinical examination, and that are not yet detectable by imaging or clinical neurological scores. This endpoint might be considered suitable for early diagnosis of stroke lesions that often occur in the Cd, but also in the context of localized brain tumors such as glioblastoma, or in Huntington disease, which is associated with death of striatal neurons^32^.

The late adverse effects on walking with a preference toward the right direction and stereotypic circling behavior six months after irradiation were concomitant with the establishment of lesions. Although the stereotypic circling behavior was alleviated twelve months after irradiation, suggesting compensation from the contralateral Cd, we observed a reversal of the walking preference toward the left direction. We postulate that the contralateral Cd was not able to fully compensate the radio-ablation of the targeted Cd, suggesting a lateralization of motor-related Cd function. A default in the mechanism of contralateral Cd compensation is also conceivable^33^.

Stereotactic ablation by ionizing radiation, *i.e*. stereotactic radiosurgery (SRS), has been extensively studied with Gamma-Knife^34,35^ and recently explored with focused ultra-sound^36^. Here, we used a hypofractionated SRT combined with synchrotron SRS (also called MRT) to target the right Cd of Yucatan minipig that yielded a focal ablation of cerebral tissue with limited effects on surrounding brain tissues as shown in previous studies^16,20,26,37,38^. MRT has been almost exclusively conducted in small animal models. By substituting one clinical SRT irradiation fraction with one fraction of bi-directional MRT, we reached higher doses than in a conventional fractionation scheme and paved the way for translational research on deep-seated targets. Indeed, we showed that our combined strategy was highly efficient in bringing about a nearly complete ablation of the targeted Cd, from 120 days to 360 days after irradiation without unexpected side effects other than the ones linked to the above-mentioned ablation. The MRI contrast enhancement was restricted to the target and is in accordance with previous MRT^16^ and gamma knife^27^ studies. Further studies are required to investigate whether the ipsilateral modifications might be attributed to either long-term and secondary consequences^27^ of the target ablation through necrosis and local inflammation processes, or by a ballistic effect in MRT (*i.e*. the use of only 2 orthogonal beams), which might required to be further optimized. *A contrario*, the contralateral hemisphere showed no radio-induced adverse effects. This might be due to the unique radio-tolerance of normal tissues to unidirectional microbeam exposures^26^. An anatomical and histopathological study of the minipig brains is in progress and the findings will be opposed to this hypothesis. This is however a whole research study in itself. All these findings encourage us to use the healthy minipig model to transfer MRT to clinics, as a boost in SRT treatment schemes for patients with brain tumors^19,20^. It could also be interesting to investigate the performances of the technique as solo SRS option for the treatment of patients with non malignant neurologic diseases such as epilepsy^16^.

## Conclusions

We provided an extensive description of the dynamic and evolving effects of SRT and MRT based radio-ablation of the right Cd in minipigs with neuroimaging and behavioral approaches. The radio-ablation was effective and induced a nearly eighty-percent volume reduction of the targeted Cd. We observed a sub-acute effect on hedonic and/or motivational behavior reverted to normal standards six months after irradiation. These changes were detected before the onset of any observable neuroradiological lesions of the targeted Cd, the latter being associated with motor disturbances. However, this late effect on motor function twelve months after irradiation might also be attributed to the impairment of the brain structures surrounding the targeted Cd. Altogether, these results suggest that the combination of hypofractionated stereotactic radiotherapy and synchrotron based radiosurgery (MRT-Boost) could be a promising approach for the radioablation of pathological brain sectors in the context of neurological disorders. Further, the emergence of disturbed eating behavior might be used to diagnose the presence of ongoing Cd lesions before they can be detected by imaging.

## Materials and Methods

### Animals and housing conditions

Experiments were conducted in accordance with the current ethical standards of the European Union (Directive 2010/63/EU), Agreement No. C35-275-32 and Authorization No. 35-88. The Regional Ethics Committee in Animal Experiment of Brittany and the ethics committee of the European Synchrotron Radiation Facility (ESRF) in Grenoble, France (ETHAX) have approved the entire procedure described in this paper (project N° 2016102410037, AP: 2016101009407362), which was further approved by the French Ministry of Research. A total of seven six-month-old 20-kg male Yucatan minipigs were used in this study: irradiated animals (n = 3), and control animals (n = 4) that were only subjected to the behavioral tests and not to the irradiation procedure and MRI. Animals were housed in two pens (400 × 300 × 80 cm^3^), with a maximum of four animals per pen. Animals had free access to water and were fed with a daily ration of minipig standard feed (from 500 g for 6-month-old animals to 800 g for 18-month-old animals). A chain was suspended in each pen to enrich the environment of the animals and fulfill their natural disposition to play. The room was maintained at 24 °C with a 13:11-h light-dark cycle.

### Anesthesia

Animals were anesthetized for irradiations and brain imaging procedures. Induction of anesthesia was performed with an intramuscular injection of ketamine (5 mg/kg – Imalgene 1000, Merial, Lyon, France) in overnight-fasted animals. Isoflurane inhalation (Aerane 100 ml, Baxter SAS, France) with a flow of 3–5% v/v and 2–3% v/v respectively was then used to uphold anesthesia, suppress the pharyngotracheal reflex and then establish a surgical level of anesthesia. After intubation, anesthesia was maintained with 3% v/v isoflurane and mechanical respiration allowed adjustment of respiratory frequency.

### Radio-ablation of the right caudate nucleus

**Figure 5:**
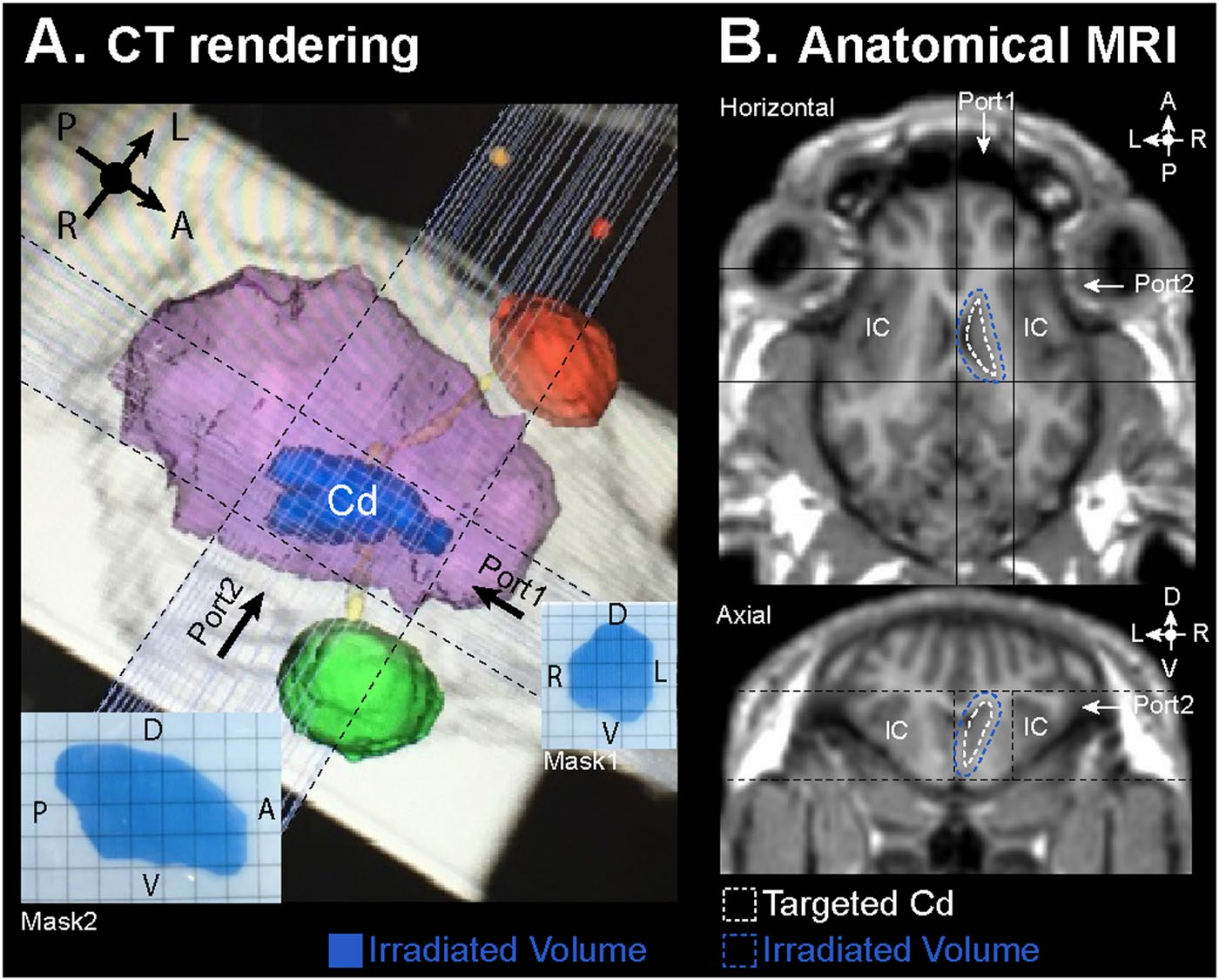
(**A**) CT volumetric rendering of the irradiated volume in blue, the animal brain volume in pink, the eye in green (right) and red (left), and the two microbeams radiotherapy (MRT) ports within the black dotted lines. Checkered insets: For each port, a radiosensitive mask that was aligned to the MRT beam is shown; spatial orientation defined by A: anterior, P: posterior, R: right, L: left, V: ventral, and D: dorsal. (**B**) Anatomical MRI visualization (axial and horizontal orientations, before Gd-DOTA injection) of the targeted caudate nucleus (Cd) edged by white dotted line and, with an additional 2-mm border, the irradiated volume in blue dotted line and the two MRT ports within the black dotted lines. IC: insular cortex of both hemispheres was on the trajectory of the MRT ports.

The radio-ablation of the right caudate nucleus (Cd) in pigs was performed by combining hypofractionated stereotactic radiotherapy (SRT)^17^ with microbeam radiation therapy (MRT). The 3rd fraction of a 3 × 11 Gy conventional hypofractionated SRT irradiation scheme (as used typically for the treatment of brain metastases) was replaced by one fraction of MRT. The SRT was performed on a Varian (Palo Alto, USA) Clinac 2100 linear accelerator (6 MV). Positioning of the pigs was adjusted using the Brainlab ExacTrac system based on CT images (Hispeed NX/I; General Electric Medical Systems, Milwaukee, WI, USA) acquired at Rennes Platform for Multimodal Imaging and Spectroscopy (PRISM, AniScans). Target volume and planning target volume (PTV) have been delineated. Two dose fractions in 3 days were then delivered according to the treatment plan. Three arcs were performed during each SRT fraction in order to deliver a minimum of 7.7 Gy to the caudate (*i.e*. 11 Gy at the isocenter in the PTV). MRT was delivered in bidirectional mode (90 degrees crossfired incidences) three days later at the ID17 beamline of the European Synchrotron Radiation Facility (ESRF) in Grenoble, France. The pigs were positioned with submillimetrical accuracy in the beam using a 6-axis goniometer and the image guidance protocol developed by Donzelli *et al*.^39^. The microbeams arrays (50-µm-wide with a center-to-center spacing of 400 µm) were defined by a multislit collimator^40^. In order to irradiate the entire PTV, the microbeams array (which is of small height) was scanned vertically over the target and through a cerrobend collimator, which gives the shape of the Cd as delineated for the treatment planning system. The constant scanning speed was calculated for delivering the required dose, by hybrid dose calculation developed for MRT^41^. Two perpendicular beams were used in order to deliver a minimal valley dose of 15 Gy at the PTV (Fig. 5).

### Magnetic resonance imaging (MRI)

Image acquisition was performed on a 1.5-T magnet (Siemens Avanto) at the PRISM imaging platform (AgroScans) 13 days before irradiation and 6, 24, 60, 120, 180 and 360 days after irradiation. Acquisitions were performed as previously described^42^ using a combination of coils (Body and Spine matrix coils) for optimized signal-to-noise ratio acquisition. *T1 weighted anatomical image acquisition*: a MP-RAGE sequence was adapted for adult minipig anatomy (1.2 × 1.2 × 1.2 mm^3^, NA = 2, TR = 2400 ms, TE = 3.62 ms, TI = 854 ms, FA = 8°, acquisition duration 15 min). *Gadolinium-based contrast enhancement for vascular wall integrity visualization*: two T1 weighted, spin echo, images (1 × 1 × 2 mm^3^, NA = 2, TR = 512 ms, TE = 12 ms, acquisition duration 4 min) were acquired before and three minutes after an intravenous injection of Gd-DOTA (100 µmol/kg; Guerbet SA, France) in an ear vein and flushed with 10 mL of saline. For the determination of vascular wall integrity, *i.e*. blood brain barrier (BBB) disruption, contrast enhancement was calculated by subtracting the T1 weighted image after Gd-DOTA injection with the T1 weighted image before injection.

Two regions of interests (ROI) were delineated on the T1 weighted anatomical images: the targeted right Cd and the contralateral structure, allowing for measurement of their respective volumes.

### Eating behavior tests

Eating behavior tests were performed 1 and 6 months after irradiation in individual slatted cages in which the animals received their feed either in one go or by pressing a button triggering the delivery of a 30-g portion, depending on the testing phase. Briefly, animals were first habituated to the testing cages for two days and then further trained three hours per day during five days to press a button triggering the feed delivery. After training, animals were evenly given additional feed in order to complete their normal daily feed ration.

#### Two-choice feed test

The two-choice test was performed three hours per day, during three consecutive days, with an operant conditioning approach^43,44^ in order to assess the animals’ preferences between the standard feed and the standard feed with added sucrose (20%). Animals were able to choose between the two different feeds, delivered in two different troughs by pressing the allocated button. After testing, animals were evenly given additional feed in order to complete their normal daily feed ration. Feeds were interchanged over days in order to avoid any laterality bias. The ratio of the number of presses for the standard feed and the number of presses for the standard feed with sucrose was computed for each day.

#### Eating microstructure test

In order to assess the eating behavior in normal meal condition, the eating microstructure test was adapted from Ochoa *et al*.^45^. During two testing days the animals were given their entire meal consisting of one kg of their standard feed, and the eating time was recorded. This time was defined either as the time necessary for the animal to finish its meal, or as the time after which the animal stopped eating for at least thirty minutes. The test lasted three hours and, after testing, the animals were evenly given additional feed in order to complete their normal daily feed ration. The average time between the two testing days was then computed.

### Openfield test

The openfield test was performed in a 5 × 5 m arena. Animals were free to explore the arena for 10 minutes. The openfield test was repeated twice in a 24-hour interval, for each of the following time points: 1, 6 and 12 months after irradiation. For locomotion assessment, the arena was virtually divided into 16 identical squares, and the number of squares crossed with at least two forepaws was recorded. Walking orientation was also recorded with continuous sampling of the animals’ trajectory and defined as walking straightforward, turning left, or turning right. This was performed to assess the potential appearance of motor lateralization after the irradiation treatment, in comparison to the control group. Data were expressed as the percentage of each trajectory type during the tests for each animal. The average value for the two consecutive testing days for each time point was used for statistical analysis.

### Statistical analysis

Data are expressed as mean ± standard error of the mean (SEM). ANOVA tests were performed with SPSS to determine statistical significance. A p-value < 0.05 was considered significant. A trend was considered with 0.05 < p < 0.10.

## Data Availability

Data presented in this paper will be deposited in an official repository (DRYAD) after publication.
